# Nuttalliellidae in Burmese amber: implications for tick evolution

**DOI:** 10.1017/S0031182024000477

**Published:** 2024-08

**Authors:** Lidia Chitimia-Dobler, Stephan Handschuh, Jason A. Dunlop, Ronel Pienaar, Ben J. Mans

**Affiliations:** 1 Department of Rickettsiology and Virology, Bundeswehr Institute of Microbiology, Munich, Germany; 2 Department of Infection and Pandemic Research, Fraunhofer Institute of Immunology, Infection and Pandemic Research, Penzberg, Germany; 3 VetCore Facility for Research / Imaging Unit, University of Veterinary Medicine, Vienna, Austria; 4 Museum für Naturkunde, Leibniz Institute for Evolution and Biodiversity Science, Berlin, Germany; 5 Epidemiology, Parasites and Vectors, Agricultural Research Council-Onderstepoort Veterinary Research, Onderstepoort, South Africa; 6 Department of Zoology and Entomology, University of the Free State, Bloemfontein, South Africa; 7 Department of Life and Consumer Sciences, University of South Africa, Pretoria, South Africa

**Keywords:** cretaceous, *Deinocroton*, fossil, *Legionaris*, Myanmar, *Nuttalliella*, Nuttalliellidae

## Abstract

Ticks are composed of 3 extant families (Argasidae, Ixodidae and Nuttalliellidae) and 2 extinct families (Deinocrotonidae and Khimairidae). The Nuttalliellidae possess one extant species (*Nuttalliella namaqua*) limited to the Afrotropic region. A basal relationship to the hard and soft tick families and its limited distribution suggested an origin for ticks in the Afrotropics. The Deinocrotonidae has been found in Burmese amber from Myanmar and Iberian amber from Spain, suggesting a wider distribution of the lineage composed of Deinocrotonidae and Nuttalliellidae. The current study describes 8 fossils from mid-Cretaceous (ca. 100 Ma) Burmese amber: 2 *Deinocroton* species (*Deinocroton bicornis* sp. nov.; *Deinocroton lacrimus* sp. nov.), 5 *Nuttalliella* species (*Nuttalliella gratae* sp. nov., *Nuttalliella tuberculata* sp. nov., *Nuttalliella placaventrala* sp. nov., *Nuttalliella odyssea* sp. nov., *Nuttalliella tropicasylvae* sp. nov.) and a new genus and species (*Legionaris* nov. gen., *Legionaris robustus* sp. nov.). The argument is advanced that *Deinocroton* do not warrant its own family, but forms part of the Nuttalliellidae comprising 3 genera, *Deinocroton*, *Legionaris* nov. gen. and *Nuttalliella*). Affinities of Burmese tick fossils to the Australasian region, specifically related to rifting of the Burma terrane from northern Australia ~150 million years ago, suggest that *Nuttalliella* had a much wider distribution than its current limited distribution. The distribution of *Nuttalliella* likely stretched from Africa over Antarctica and much of Australia, suggesting that extant members of this family may still be found in Australia. Considerations for the geographic origins of ticks conclude that an Afrotropic origin can as yet not be discarded.

## Graphical abstract

**Figure:**
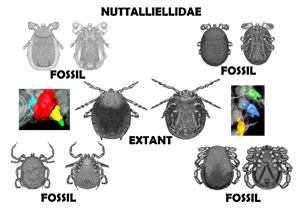

## Key findings

- The genus *Nuttalliella* is reported from Cretaceous Burmese amber.
- It represents the oldest (and only) fossil records of this genus.
- Interestingly, the extant family is restricted to Africa.
- The Nuttalliellidae comprises the genera *Nuttalliella*, *Deinocroton* and *Legionaris* nov. gen.
- Implications of a Gondwanan origin for these fossils and for tick origins are discussed.

## Introduction

Ticks (Arachnida: Parasitiformes: Ixodida) are economically important haematophagous ectoparasites of vertebrates (Sonenshine and Roe, 2013). Extant families include the hard ticks (Ixodidae, *n* ~ 731 species), the soft ticks (Argasidae, *n* ~ 216 species) and the Nuttalliellidae, a monotypic family with one species, namely *Nuttalliella namaqua* Bedford, 1931 (Dantas-Torres, 2018). Two extinct families only found as amber fossils are also known, namely the Deinocrotonidae (2 species) and the monotypic Khimairidae (Peñalver *et al*., 2017; Chitimia-Dobler *et al*., 2022).

Amber fossils have made important contributions to our understanding of tick evolution, not only indicating that lineages existed that are now extinct but also extending the minimum ages of various extant genera back to at least the cretaceous (~100 million years ago) (Mans, 2022, 2023). Extinct genera include *Deinocroton* Peñalver *et al*., 2017 that belongs to a unique family, the Deinocrotonidae (Peñalver *et al*., 2017), *Khimaira* Chitimia-Dobler *et al*., 2022 that belongs to a unique family, the Khimairidae (Chitimia-Dobler *et al*., 2022), *Cornupalpatum* Poinar and Brown, 2003 and *Compluriscutula* Poinar and Buckley, 2008 that belongs to the Metastriata (Poinar and Brown, 2003; Poinar and Buckley, 2008). Extant hard tick genera found in amber include *Ixodes* Latreille, 1795, *Archaecroton* Barker and Burger, 2018; *Amblyomma* CL Koch, 1844, *Bothriocroton* Keirans, King and Sharrad, 1994, and *Haemaphysalis* (*Allocereae*) CL Koch, 1844 (Chitimia-Dobler *et al*., 2022, 2023) and for soft ticks, *Alectorobius* Pocock, 1907 (Poinar, 1995; Klompen and Grimaldi, 2001). The oldest fossils for these genera all derive from Burmese amber (ca. 100 million years), except for *Alectorobius jerseyi* Klompen and Grimaldi, 2001 from New Jersey amber dated to ca 94–96 million years (Klompen and Grimaldi, 2001) and *Alectorobius antiquus* (Poinar, 1995) that date from Dominican amber (ca 20–15 million years).

*Nuttalliella namaqua* was considered an enigmatic species and ‘missing link’ since it presented features shared between both hard and soft tick families (Bedford, 1931). Notably, a leathery integument similar to that observed in argasids, while a terminal gnathosoma and pseudoscutum resembled similar structures observed in ixodids. However, Schulze (1935) and Aragão (1936) seemingly independently raised the genus to family level based on its unique characters, such as ball-and-socket joints and the fact that both argasid and ixodid features were found in this lineage. Biological characters are distinctly argasid in nature, with larvae showing prolonged feeding periods, while females feed fast and lay small egg batches (Mans *et al*., 2011, 2012). However, even in biology the *Nuttalliella* shows unique characters such as secretion of excess blood meal-derived water *via* the Malpighian tubules and the anal pore (Mans *et al*., 2011). Phylogenetic analysis placed the Nuttalliellidae basal to the hard and soft ticks (Mans *et al*., 2012, 2019, 2021), and it was suggested that characters unique to either hard or soft ticks, but present in the Nuttalliellidae were present in the ancestral tick lineage as well (Mans *et al*., 2016). The extinct Deinocrotonidae was suggested to be a sister group to the Nuttalliellidae since they share a leathery integument and a peudoscutum, although the former seem to lack ball-and-socket joints (Peñalver *et al*., 2017). While several *Deinocroton* fossils species have been described (Peñalver *et al*., 2017; Chitimia-Dobler *et al*., 2022), no fossils for the Nuttalliellidae have been found yet. The current study describes *Nuttalliella* fossils from Burmese amber and consider taxonomic and systematic implications for the Deinocrotonidae and the Nuttalliellidae. It also expands the Nuttalliellidae family by adding another 2 genera to the family.

## Materials and methods

### Amber specimens

The 8 specimens originate from the collections of the first author and has been deposited in the Museum für Naturkunde, Berlin. Burmese amber originates from deposits in the Hukawng Valley of northern Myanmar and has been dated to a Cenomanian age of about 100 Ma (Shi *et al*., 2012; Smith and Ross, 2018). Details about the fossil setting and specifically arachnids have been summarized in a number of studies (Grimaldi *et al*., 2002; Ross *et al*., 2010; Selden and Ren, 2017). A list of Burmese amber inclusions has been updated recently (Ross, 2022).

### Photography and computer tomography

For photography, A Keyence VHX-900F Microscope (Keyence Itasca, IL, USA) was used at 50× to 200× magnification. Helicon Focus 6.7.1 was used to combine image stacks. For computed tomography (micro-CT), specimens were mounted on a specimen holder and scanned using a Zeiss Xradia MicroXCT-400 (Carl Zeiss X-Ray Microscopy, Pleasanton, CA, USA) at 40 kVp tube voltage and 114 μA current using the 4X detector assembly. Depending on specimen size, isotropic voxel size in reconstructed volumes ranged between 1.40 μm and 2.90 μm. Reconstructed image volumes were processed and visualized by volume rendering and manual segmentation-based surface rendering using the 3D software package Amira 2022.1 (FEI SAS, a part of. Thermo Fisher Scientific). Drawings were prepared with a *camera lucida* attachment on a Leica M205C stereomicroscope (Leica Microsystems, Wetzlar, Germany), again using a combination of incident and transmitted light where appropriate. Artist's impressions were made from a combination of photographs and micro-CT scans.

### Extant tick specimens

To improve the interpretation of leg joint morphology, one member of the extant tick families Ixodidae and Nuttalliellidae were analysed. One fresh *Ixodes ricinus* (Linnaeus, 1758) specimen was fixed in 70% ethanol, and subsequently stained with 0.1% (w/v) Lugol's iodine (I_2_KI) solution for 24 h to improve soft tissue contrast. After staining, it was rinsed with distilled water and mounted in a heat-sealed pipette tip in 1.5% low melt agarose. One *Nuttalliella namaqua* specimen, which has been preserved in 70% ethanol for 7 years, was also stained with Lugol's iodine solution. Due to the long specimen storage, stain uptake was impaired. Thus, the total staining duration was 9 days in 0.1% (w/v) I_2_KI followed by 10 days in 0.25% (w/v) I_2_KI. After staining, it was rinsed with distilled water and mounted in a heat-sealed pipette tip in distilled water. Both specimens were scanned using an Xradia MicroXCT-400 (Carl Zeiss X-Ray Microscopy, Pleasanton, CA, USA) at 60 kVp/133 μA. The *I. ricinus* specimen was scanned with the 10X detector assembly at 1.06 μm voxel size, and the *N. namaqua* specimen was scanned with the 4X detector assembly at 1.99 μm voxel size. In addition to whole-body scans of the 2 species, a high-resolution interior tomography scan of the coxa, trochanter and femur of the third leg of *N. namaqua* was acquired using the 40X detector assembly at 40 kVp/200 μA and an isotropic voxel size of 0.52 μm. Reconstructed image volumes were processed and visualized by volume rendering and manual segmentation-based surface rendering using the 3D software package Amira 2022.1 (FEI SAS, a part of. Thermo Fisher Scientific).

## Results

### Systematic palaeontology for the Nuttalliellidae Class Arachnida Lamarck, 1801 Order Parasitiformes Reuter, 1909 Suborder Ixodida Leach, 1815 Family Nuttalliellidae Schulze, 1935

*Diagnosis for the Nuttalliellidae:* The presence of a pseudoscutum, a ‘soft’ integument formed by convoluted infoldings that may resemble rosettes, the sub-terminal position of the basis capitulum, palpal segment II and IV arising from the anterior segments and leg joints with deep articulation within the joint, giving them a ‘ball-and-socket’ appearance.

### Systematic palaeontology for the Nuttalliella *Nuttalliella* Bedford, 1931

*Diagnosis:* Classical ball-and-socket articulation with a round distal region going into the socket with deep condyles. Palps have a triangular aspect in dorsal view. Palpal segment II massive with segments III and IV arising from the anterior segments. Long, slender beaded legs. Genital aperture a transverse slit (external margins of lips dentate in male) among coxae coxae I, coxae II or coxa II-III.

*Type species*: *Nuttalliella namaqua* Bedford, 1931.

*Type depository*: The Iziko South African Museum (Cape Town) according to Bedford, 1931.

### *Nuttalliella odyssea* Chitimia-Dobler, Dunlop and Mans sp. nov.

http://zoobank.org/urn:lsid:zoobank.org:act:A2062BBA-6AEE-4EE0-ACBA-BA824A38D17C

*Etymology*: From the latin *oddyssea* (Odyssey from the Greek epic poem from Homer) to describe the long journey of this species from the apparent origin of this family in the Afrotropic region of Gondwana.

*Material*: Holotype B-4863 (Berlin Museum). Burmese amber, Myanmar, Late Cretaceous (Cenomanian) (Supplementary video S1).

*Diagnosis*: Position of the genital aperture among coxae II, anal groove round closing median posterior, ventral plate absent, dorsal marginal groove absent and coxae I–IV rectangular shaped.

**Figure 1.**
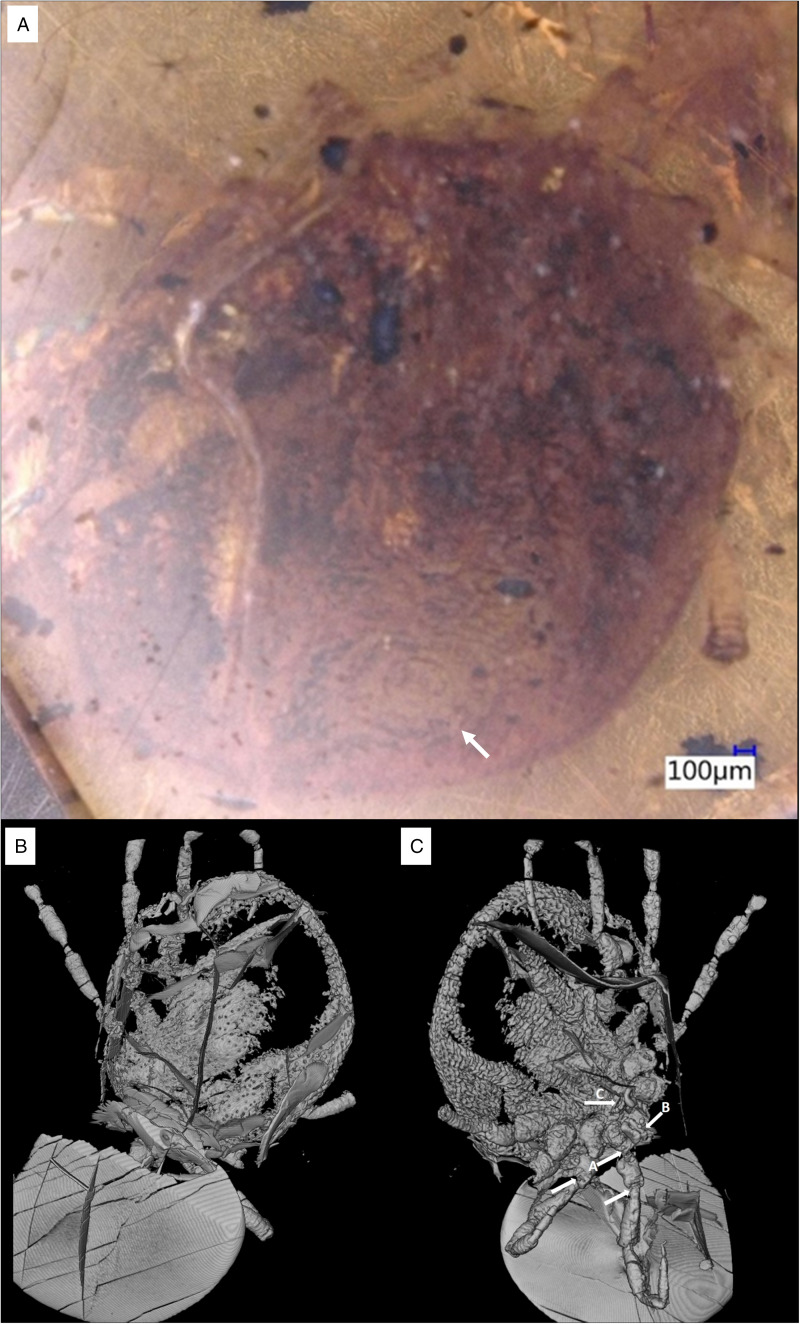
Holotype of *Nuttalliella odyssea* sp. nov., collection no. B-4863, from Late Cretaceous (ca. 100 Ma) Burmese amber from Myanmar. (A) Light microscope, arrow – anal groove. (B) Micro-CT scan of dorsal view. (C) Micro-CT scan of ventral view: A-leg joints; B-palp; C-genital aperture.

**Figure 2.**
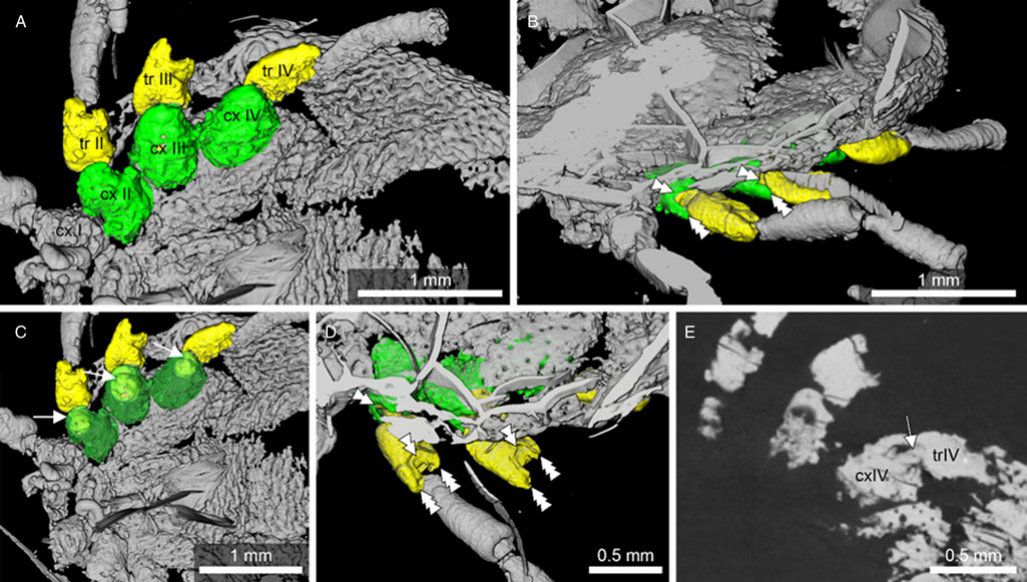
Micro-CT scan depicting the ball-and-socket joint structure found in *Nuttalliella odyssea* sp. nov. (B-4863). (A) Detail of ball-and-socket joint of trochanter inserting into the coxae of B-4863. (B) Detail of ball-and-socket joint of trochanter of B-4863. (C, D, E) Depicted are various views of coxae II, III and IV that depict the ball-and-socket joint structure found B-4863. Both the coxae and trochanter show distinct notches (double arrowheads) framed by cuticular protrusions (triple arrowheads), which constrains the movement of this joints to a single plane (like in extant *Nuttalliella namaqua*, Fig. 13).

*Description of the holotype female (B-4863)*: capitulum sub-terminal, marginal groove absent, genital aperture a transverse slit, ventral plate absent and legs with ball-and-socket joints (Figs 1 and 2, Figure S1; Supplementary video S1).

*Idiosoma*: body outline sub-circular 3.93 mm length and 3.83 mm width, dark brown, pseudoscutum could not be observed due to damage. Marginal groove absent. On venter, integument convoluted, forming closely spaced pits surrounded by elevated rosettes (Figs 1A–C).

Genital aperture a transverse slit, in a broadly oval area, median, between coxa II. Anus close to the posterior body margin, anal valves well defined, round anal groove surrounding at a slightly distance the anus closing median posterior.

**Figure 3.**
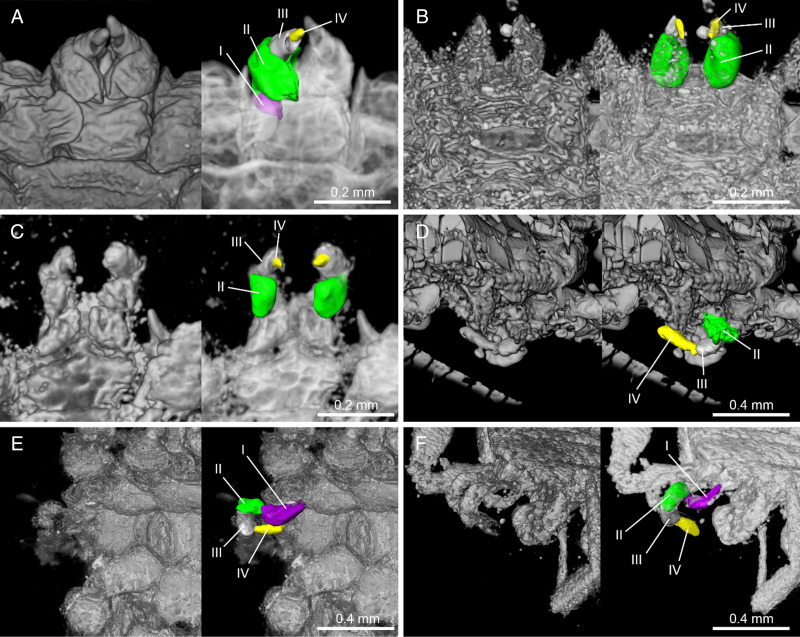
Comparison of palpal segments from various fossils. (A) *Nuttalliella namaqua* (extant species), (B) *Nuttalliella placaventrala* sp. nov. (B-4862), (C) *Nuttalliella tropicasylvae* sp. nov. (B-7243), (D) *Nuttalliella odyssea* sp. nov. (B-4863), (E) *Legionaris robustus* sp. nov. (B-4891) and (F) *Deinocroton bicornis* sp. nov. (B-4839). Palpal segments are indicated as I (violet), II (green), III (grey) and IV (yellow).

*Capitulum*: capitulum is only partly seen ventral. Palps were broken: right side segment II partly visible, segment III 0.151 mm, arises from the segment II, segment IV 0.294 mm arises from the segment III (Fig. 3D).

*Legs*: Coxae I-IV have rectangular shape. Coxae I are laterally close to the basis capituli with an anterior-posterior orientation. Coxa I right 0.512 mm length and 0.364 width. Coxae II partly contiguous to coxa I, while coxae II and III and coxae III and IV each slightly separate by an integument area. Leg segments articulate by ball-and-socket joints (Fig. 2). Only the right leg is complete, the others are broken and a different number of segments can be observed.

*Chaetotaxy*: Some spaced pits associated with seta.

### *Nuttalliella placaventrala* Chitimia-Dobler, Dunlop and Mans sp. nov.

*Etymology*: From the latin placa for plate and ventralis for ventral, referring to the distinct ventral plate observed in this fossil.

*Material*: Holotype B-4862 (Berlin Museum). Burmese amber, Myanmar, Late Cretaceous (Cenomanian) (Supplementary video S2).

*Diagnosis*: Position of the genital aperture between coxa I, anal groove curved shape, margins parallel reaching the posterior body margin, distinct large ventral plate extends from the posterior level of coxa III, below the genital aperture, widest before anal groove, reaching the posterior body margin, dorsal marginal groove indistinct if present.

**Figure 4.**
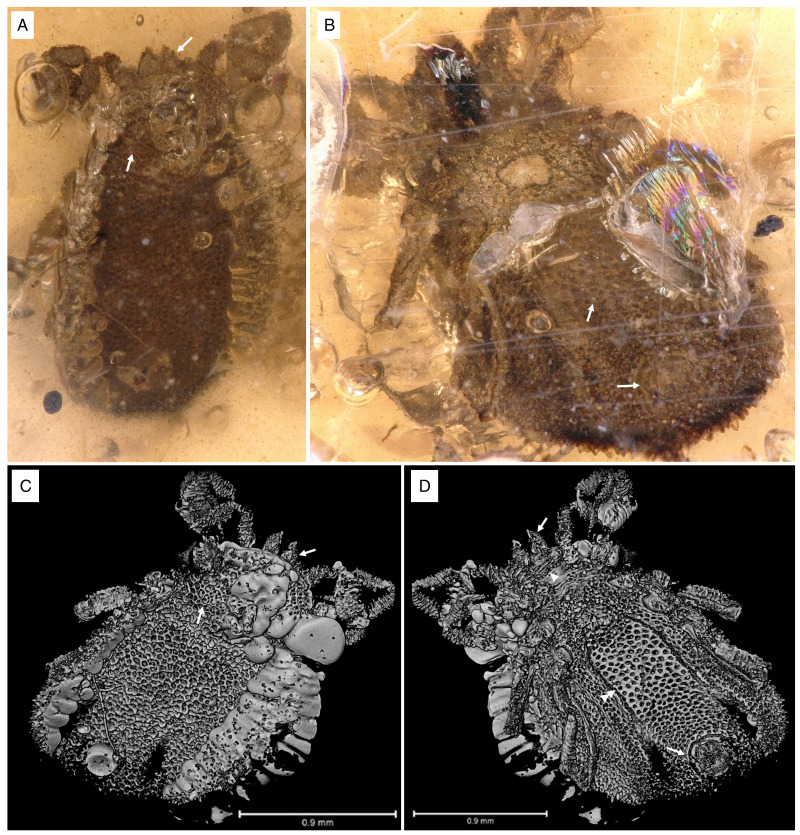
Holotype of *Nuttalliella placaventrala* sp. nov., collection no. B-4862, from Late Cretaceous (ca. 100 Ma) Burmese amber from Myanmar. (A) Dorsal view, arrow – palps and pseudoscutum. (B) Ventral view, arrow – ventral plate and anal groove. (C) Micro-CT scan, dorsal view, arrow – palps and pseudosutum. (D) Micro-CT scan, ventral view: single arrowhead – genital aperture, double arrowhead – ventral plate, arrows – palps and anal groove.

*Description of holotype male (B-4862)*: capitulum sub-terminal, genital aperture a transverse slit, ventral plate extends from the posterior level of coxa III, reaching the posterior body margin legs with ball-and-socket joints, eyes absent, lateral margin higher than the middle of the alloscutum (Fig. 4, Fig. S1; Supplementary video S2).

*Idiosoma*: body outline oval 2.01 mm length and 1.33 mm width, dark brown, pseudoscutum 0.626 mm length and 0.755 mm width, large V shape reaching the lateral and anterior margin of body; integument surface with elevation forming a network of irregular shallow larger compartments (Fig. 4A). Eyes absent. Integument extremely convoluted, forming closely spaced pits surrounded by elevated rosettes, dorsally and ventrally. Marginal groove could not be observed. Dorsally, the lateral margin is 0.122 mm higher than the middle of the alloscutum (Fig. 4B).

sGenital aperture a transverse slit, between coxa I (Fig. 4C). Ventral plate extends from the posterior level of coxa III, below the genital aperture, widest before anal groove, 0.489 mm, reaching the posterior body margin. Anus close to the posterior body margin; anal valves well defined, anal groove slightly anterior the anus, curved shape, margins parallel reaching the posterior body margin.

*Capitulum*: basis capituli not visible dorsal, palps: segment I recessed and thus not visible, segment II massive (0.143 mm long), segment III smaller (0.050 mm), arising from segment II, segment IV (0.063 mm), arising from segment III (Fig. 3B).

*Legs*: Coxae I are laterally close to the basis capituli with an anterior–posterior orientation. Coxae II partly contiguous to coxa I, while coxae II and III and coxae III and IV each slightly separate by an integument area. Leg segments are articulated by a ball-and-socket joints.

*Chaetotaxy*: Very few setae visible on the first legs, palps and some spaced pits associated with seta.

### *Nuttalliella tuberculata* Chitimia-Dobler, Dunlop and Mans sp. nov.

http://zoobank.org/urn:lsid:zoobank.org:act:ACAD75F3-7D6D-43CD-B7F2-1F6A20C6A64B

*Etymology*: From the latin tuberculatus, referring to the small rounded projections found on the integument and legs that is distinct feature in this species.

*Material*: Holotype B-4925 (Berlin Museum). Burmese amber, Myanmar, Late Cretaceous (Cenomanian) (Supplementary video S3).

*Diagnosis*: Position of the genital aperture between coxa II and III, anal groove pore slightly visible anterior to the anus, curved shape, reaching the posterior body margin, ventral plate not visible, dorsal marginal groove slightly distinct.

**Figure 5.**
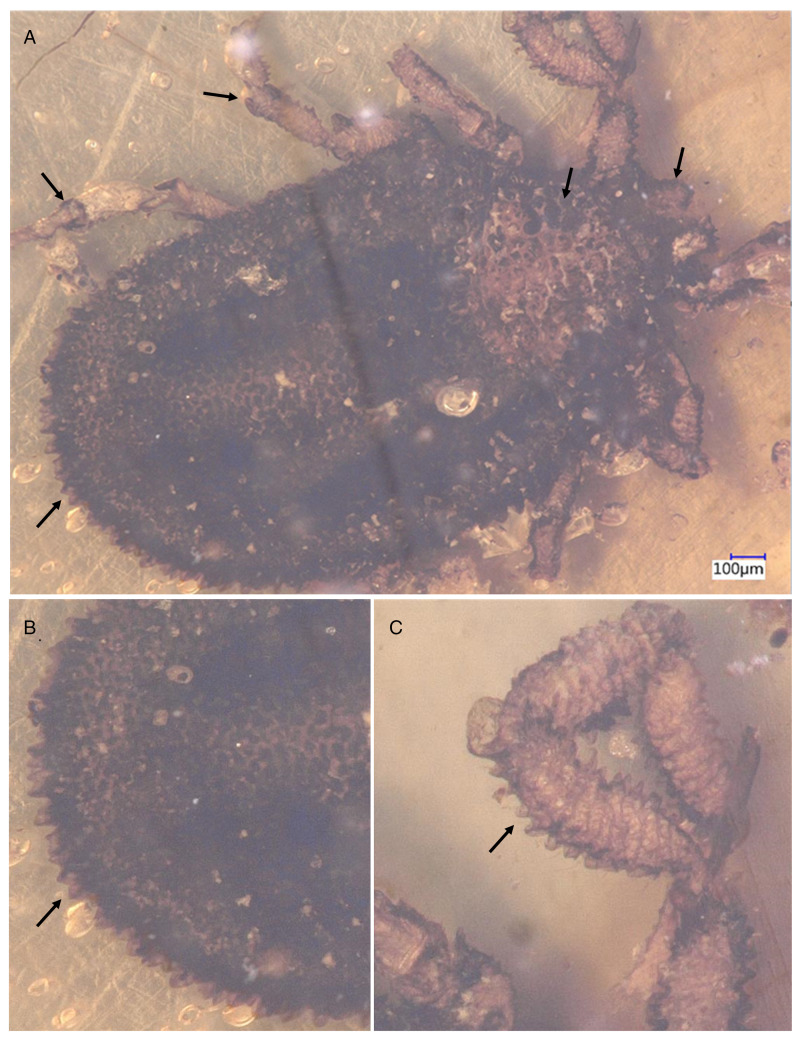
Holotype of *Nuttalliella tuberculata* sp. nov., collection no. B-4925, from Late Cretaceous (ca. 100 Ma) Burmese amber from Myanmar. (A) Light microscopy – arrows indicate palps, pseudoscutum, leg joints and integumental protrusions. (B) Close up of integumental protrusions. (C) Close up of protrusions on legs.

*Description of the holotype male (B-4925)*: capitulum sub-terminal, genital aperture a transverse slit, ventral plate absent, legs with ball-and-socket joints, eyes absent, small rounded projections on the integument, marginal groove slightly distinct (Fig. 5).

*Idiosoma*: body outline oval 1.95 mm length and 1.20 mm width, dark brown, pseudoscutum 0.34 mm length and 0.46 mm width, large V shape reaching the lateral and anterior margins of body; integument surface with elevation forming a network of irregular shallow larger compartments or protrusions/tubercules. Eyes absent. Integument extremely convoluted, forming closely spaced pits surrounded by elevated rosettes, dorsally and ventrally. Dorsally, the lateral margin is 0.21 mm higher than the middle of the alloscutum, with a slightly defined marginal groove.

Genital aperture a transverse slit, between coxa II and III. Ventral plate not visible. Anus close to the posterior body margin; anal groove slightly visible anterior to the anus, curved shape, reaching the posterior body margin.

*Capitulum*: basis capituli not visible dorsal, palp segments could not be measured due to bad specimen preservation.

*Legs*: Coxae I are laterally close to the basis capituli with an anterior-posterior position. Coxae II partly contiguous to coxa I, while coxae II and III and coxae III and IV each slightly separate by an integument area. Legs also have protrusions.

*Chaetotaxy*: not visible

### *Nuttalliella gratae* Chitimia-Dobler, Dunlop and Mans sp. nov.

http://zoobank.org/urn:lsid:zoobank.org:act:46CB6C08-6CCE-4B23-93F5-20A3270C3708

*Etymology*: From the latin for grateful, referring to our good fortune that this fossil survived the ravages of time, given its advanced state of disintegration.

*Material*: Holotype B-4927 (Berlin Museum). Burmese amber, Myanmar, Late Cretaceous (Cenomanian) (Supplementary video S4).

*Diagnosis*: Position of the genital aperture: between coxa II, anal groove converging and closing, not reaching the posterior body margin, ventral plate extends from the posterior of coxa II, diverging posteriorly and not reaching the posterior body margin, dorsal marginal groove well-defined.

**Figure 6.**
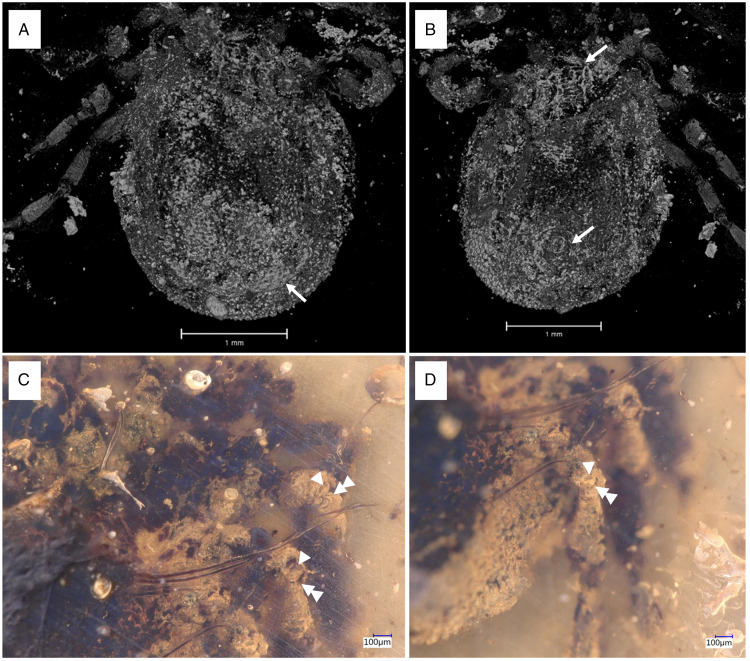
Holotype of *Nuttalliella gratae* sp. nov., collection no. B-4927, from Late Cretaceous (ca. 100 Ma) Burmese amber from Myanmar. (A) Micro-CT scan of dorsal view, arrow – marginal groove. (B) Micro-CT scan of ventral view, arrow – anal groove. (C+D) Light microscopy to show detail of leg joints. Single arrowhead indicate the ball region, while double arrowheads indicate the neck region.

*Description of the holotype male (B-4927)*: capitulum sub-terminal, genital aperture a transverse slit, dorsal lateral margin higher than the alloscutum, well-defined marginal groove, legs with ball-and-socket joints, eyes absent (Fig. 6).

*Idiosoma*: body outline oval 3.02 mm length and 2.31 mm width, dark brown, pseudoscutum only partly seen; integument surface with elevation forming a network of irregular shallow larger compartments. Eyes absent. Integument extremely convoluted, forming closely spaced pits surrounded by elevated rosettes, dorsally and ventrally. Dorsally, the lateral margin is 0.18 mm higher than the middle of the alloscutum, with a well-defined marginal groove (Fig. 6A and B).

Genital aperture a transverse slit, between coxa II (Fig. 6A). Ventral plate extends from the posterior of coxa II, diverging posteriorly and no reaching the posterior body margin. Anus distant from the posterior body margin; anal valves well defined, anal groove slightly anterior the anus, converging and closing, not reaching the posterior body margin.

*Capitulum*: basis capituli not visible dorsal, palps not clearly visible due to organic dirt in the amber and lack of contrast in micro-CT.

*Legs*: Coxae I are laterally close to the basis capituli with an anterior–posterior orientation. Coxae II partly contiguous to coxa I, while coxae II and III each slightly separated by an integument area, right. Coxa IV right and coxae III and IV left missing. Leg segments are articulated by ball-and-socket joints (Fig. 6C and D).

*Chaetotaxy*: Some spaced pits associated with setae.

### *Nuttalliella tropicasylvae* Chitimia-Dobler, Dunlop and Mans sp. nov.

http://zoobank.org/urn:lsid:zoobank.org:act:BF61E73E-52B9-41D8-814F-D96201DECDB0

*Etymology*: From the latin ‘tropica sylva’ for tropical forest, referring to the presence of this genus in the primeval tropical amber forest, an unusual ecological niche for extant members of this genus.

*Material*: Holotype B-7243 (Berlin Museum). Burmese amber, Myanmar, Late Cretaceous (Cenomanian) (Supplementary video S5).

*Diagnosis*: Position of the genital aperture between coxa II, anal groove converging posteriorly and reaching the posterior body margin, ventral plate extends posterior of coxa IV diverging posteriorly, widest before anal groove, dorsal marginal groove absent.

**Figure 7.**
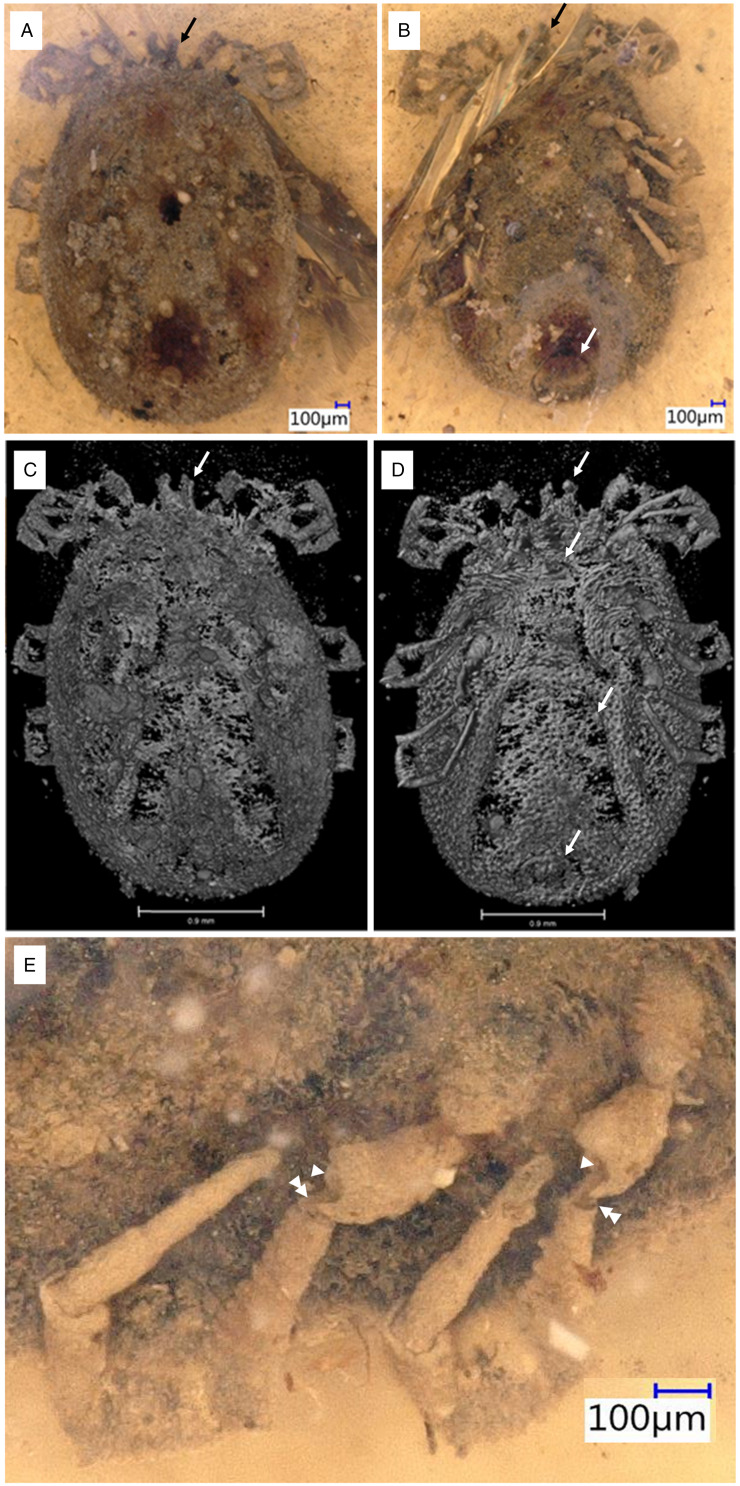
Holotype of *Nuttalliella tropicasylvae* sp. nov., collection no. B-7243, from Late Cretaceous (ca. 100 Ma) Burmese amber from Myanmar. (A/B) Dorsal: arrow – palps and ventral: arrow – palps, anal groove. (C/D) Micro-CT scan of dorsal: arrow – palps and ventral: arrow – palps, anal groove and ventral plate. (E) Detail of leg joints. Single arrowheads indicate the ball region, while double arrowheads indicate the neck region.

*Description of the holotype male (B-7243)*: capitulum sub-terminal, genital aperture a transverse slit, ventral plate extends posterior of coxa IV, dorsal lateral margin higher than the alloscutum, without a marginal groove, legs with ball-and-socket joints, eyes absent (Fig. 7).

*Idiosoma*: body outline oval 2.84 mm length and 2.09 mm width, dark brown, pseudoscutum 0.49 mm length and 0.91 mm width, large V shape reaching the lateral and anterior margin of body; integument surface with elevation forming a network of irregular shallow larger compartments. Eyes absent. Integument extremely convoluted, forming closely spaced pits surrounded by elevated rosettes, dorsally and ventrally. Dorsally, the lateral margin is 0.24 mm higher than the middle of the alloscutum, without a marginal groove (Fig. 7A and C).

Genital aperture a transverse slit, between coxa II (Fig. 7B and D). Ventral plate extends posterior of coxa IV diverging posteriorly, widest before anal groove, 1.16 mm, not reaching the posterior margin. Anus close to the posterior body margin; anal valves well-defined, anal groove slightly anterior the anus, converging posteriorly and reaching the posterior body margin (Fig. 7B and D).

*Capitulum*: basis capituli not visible dorsal, palps with 4 segments: segment I recessed, not visible dorsally, segment II massive 0.155 mm long, segment III smaller 0.093 mm, arising from the segment II, segment IV 0.025 mm, arising from the segment III (Fig. 3C).

*Legs*: Coxae I are laterally close to the basis capituli with an anterior-posterior position. Coxae II partly contiguous to coxa I, while coxae II and III and coxae III and IV each largely separate by an integument area. Leg segments are articulate by a ball-and-socket joints (Fig. 7E).

*Chaetotaxy*: Some spaced pits associated with setae.

### Differences between the described Nuttalliella species

*Nuttalliella* species differed in body size, palps segments size and position of the genital aperture: between coxa II *N. odyssea*, between coxa I in *N. placaventrala*, between coxa II *N. tropicasylvae* and *N. gratae*, between coxa II-III, *N. tuberculata*. The anal groove slightly anterior of the anus is also different: round closing median posterior for *N. odyssea*; curved shape, margins parallel reaching the posterior body margin for *N. placaventrala*; slightly visible anterior to the anus, curved shape, reaching the posterior body margin *N. tuberculata*; converging and closing, not reaching the posterior body margin *N. gratae*; converging posteriorly and reaching the posterior body margin *N. tropicasylvae*. Ventral plate is also different: absent in *N. odyssea*; extends from the posterior level of coxa III, below the genital aperture, widest before anal groove, reaching the posterior body margin *N. placaventrala*; not visible *N. tuberculata*; extends from the posterior of coxa II, diverging posteriorly and not reaching the posterior body margin *N. gratae*; extends posterior of coxa IV diverging posteriorly, widest before anal groove *N. tropicasylvae*. The dorsal marginal groove could also be considered in the differential diagnostic: absent in *N. odyssea*; could not be observed due to the artefacts *N. placaventrala*; slightly distinct *N. tuberculata*; well-defined marginal groove *N. gratae*; marginal groove absent *N. tropicasylvae*.

The possibility that the female *N. odyssea* may the same species as one of the male fossils is possible. However, no strong evidence indicates any affinity of the female to any of the males described. It would as such be more prudent to retain a novel species until stronger evidence may be found, for example ticks mating or associating in amber.

#### Systematic palaeontology for Legionaris Class Arachnida Lamarck, 1801 Order Parasitiformes Reuter, 1909 Suborder Ixodida Leach, 1815 Family Nuttalliellidae Schulze, 1935 *Legionaris* Chitimia-Dobler, Dunlop and Mans gen. nov.

http://zoobank.org/urn:lsid:zoobank.org:act:A75A61C9-4D4E-4F6A-B76F-4EC7E5B82E15

*Diagnosis:* Leg articulation as condyles joints with an ovoid distal region. Massive palpal segment I with segments II, III and IV arising from the anterior segments. Segment III bent direct on the arising point from the segment II. Very robust legs. Genital aperture bipartite in appearance, with bulging triangular forms and a narrow central flat line between, flanked around by an oval groove, located to the posterior level of coxa I.

*Type species*: *Legionaris robustus* Chitimia-Dobler, Dunlop and Mans sp. nov.

*Type depository*: Berlin Museum (Holotype B-4891) as designated in this study.

#### *Legionaris robustus* Chitimia-Dobler, Dunlop and Mans sp. nov.

http://zoobank.org/urn:lsid:zoobank.org:act:7EAD1F64-2543-43BD-8358-935CAC8FBF26

*Etymology*: The genus name *Legionaris* refers to an ever-expanding list of genera and species that belong to the larger Nuttalliellidae family. The species name *robustus* refers to the robust legs of this species that differentiate it from all other Nuttallielliedae thus far described.

*Material*: Holotype B-4891 (Berlin Museum). Burmese amber, Myanmar, Late Cretaceous (Cenomanian) (Supplementary video S6).

*Diagnosis*: Leg articulation as condyles joints with an ovoid distal region. Massive palpal segment I with segments II, III and IV arising from the anterior segments. Segment III bent direct on the arising point from the segment II. Very robust legs. Genital aperture bipartite in appearance, with bulging triangular forms and a narrow central flat line between, flanked around by an oval groove, located to the posterior level of coxa I.

**Figure 8.**
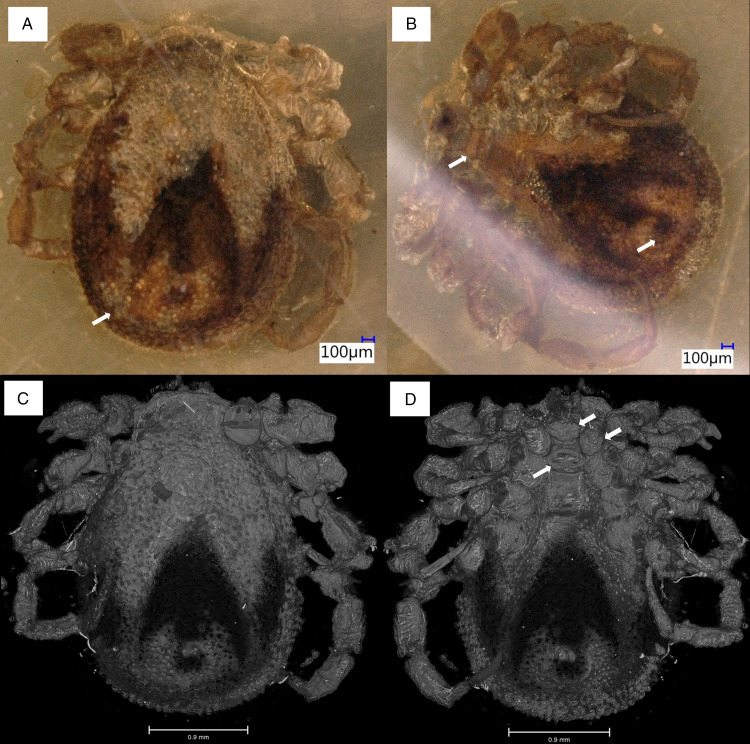
Holotype of *Legionaris robustus* sp. nov., collection no. B-4891, from Late Cretaceous (ca. 100 Ma) Burmese amber from Myanmar. (A) Dorsal view, arrow – marginal groove. (B) Ventral view, arrow – genital aperture and anal groove, coxae. (C) Dorsal micro-CT scan. (D) Ventral micro-CT scan. Arrows mark the base capituli, genital aperture, coxae and anal groove.

*Description of the holotype male (B-4891)*:

*Idiosoma*: body subtriangular 3.04 mm length and 2.06 mm width, pseudoscutum 0.28 mm length and 0.80 mm width, V shape reaching marginal groove; integument surface with few irregular shallow large compartments. Integument surface with regular shallow large compartments, dorsally and ventrally. Dorsally, with well-defined marginal groove, all around the dorsal surface (Fig. 8A and C). Eyes absent.

Genital aperture bipartite in appearance, with bulging triangular forms and a narrow central flat line between, flanked around by an oval groove, to the posterior level of coxa I (Fig. 8B and D). Marginal ventral groove from the coxae IV. Ventral plate extends from the level of coxa III, diverging posteriorly, widest to the anus level, 0.99 mm, reaching the marginal groove. Anus distant from the posterior body margin; anal valves well-defined, anal groove slightly anterior to the anus, encircle the anus and close in a V shape slightly anterior the marginal groove.

*Capitulum*: basis capituli not dorsally visible (sub-terminal), rectangular ventral, palps robust: segment I massive 0.244 mm, segment II smaller 0.144 mm, arises from the segment I and anteriorly divided in 2, like 2 condyles, segment III 0.182 mm, arises between the condyles of segment II, bended, distally ends form 2 small condyles and ventrally a narrow formation connects with segment IV, segment IV 0.181 mm, arises from the segment III, much thinner that the other segments (Fig. 3E).

**Figure 9.**
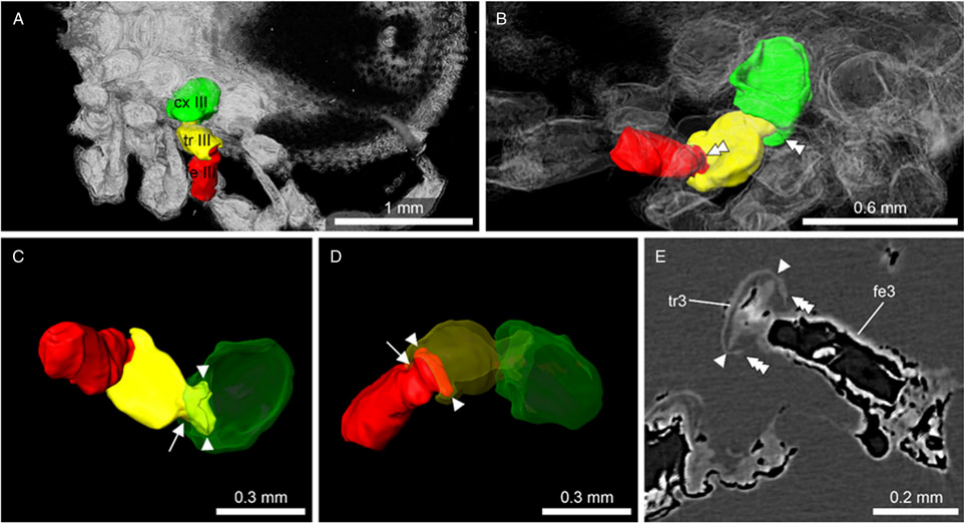
Detail of the ball-and-socket-like joints of *Legionaris robustus* sp. nov., collection no. B-4891. (A) Coxae III, trochanter III and femur III are highlighted in green, yellow and red, respectively. (B) External appearance of the ball-and-socket joint of the coxae-trochanter-femur. (C) Internal structure of the ball-and-socket joints between the coxae-trochanter. Condyles are indicated. (D) Internal structure of the ball-and-socket joints between the trochanter-femur. Condyles are indicated. (E) Micro-CT scan to show the condyles in unmodeled data. Single arrowheads indicate the condyles and triple arrowheads the rim, arrows indicate the neck region of trochanter and femur.

*Legs*: all leg segments are robust. Coxae rectangular, without spurs. Coxae I apical inner edge contiguous to basis capituli. Coxae II, III and IV contiguous to each other's. Coxae and trochanter and trochanter femur joints are articulated by a rectangular form with a well-defined hole in the middle and socket joint (Fig. 9).

*Chaetotaxy*: Some spaced pits associated with setae.

#### Systematic palaeontology for Deinocroton Class Arachnida Lamarck, 1801 Order Parasitiformes Reuter, 1909 Suborder Ixodida Leach, 1815 Family Nuttalliellidae Schulze, 1935 Deinocroton Peñalver *et al*., 2017

*Diagnosis:* Leg articulation as condyles with a round–ovoid distal region. Palps very long, segments III and IV arise from the anterior segments; segment II bent as an arch; segment IV almost reach the basis capituli. Legs long and strongly flattened laterally from trochanters to tarsi and sometimes with transverse ridges on some all or some leg segments. Transverse genital aperture between coxae II on male and between coxae II and III on female (according to Peñalver *et al*., 2017).

*Type species*: *Deinocroton draculi* Peñalver *et al*., 2017

*Type depository*: American Natual History Museum as designated by (Holotype B-4891) as designated by Peñalver *et al*. (2017).

#### *Deinocroton bicornis* Chitimia-Dobler, Dunlop and Mans sp. nov.

http://zoobank.org/urn:lsid:zoobank.org:act:A326BCD2-CE75-4E8B-BE7D-A37B428F64F2

*Etymology*: *Deinocroton* derive from the genus name from Greek *deinos*, ‘terrible’ and *krotṓn* ‘tick’ (Peñalver *et al*., 2017), while *bicornis* refer to the 2 spurs present on coxae I that differentiate it from other *Deinocroton* species described thus far.

*Material*: Holotype B-4839 (Berlin Museum). Burmese amber, Myanmar, Late Cretaceous (Cenomanian) (Supplementary video S7).

*Diagnosis*: Leg articulation as condyles with a round–ovoid distal region. Palps very long, segments III and IV arise from the anterior segments. Segment II bent as an arch. Segment IV almost reach the basis capituli. Legs long and strongly flattened laterally from trochanters to tarsi, with few small transverse ridges on some leg segments. Transverse genital aperture between coxae II.

*Description of the holotype male (B-4839):*

**Figure 10.**
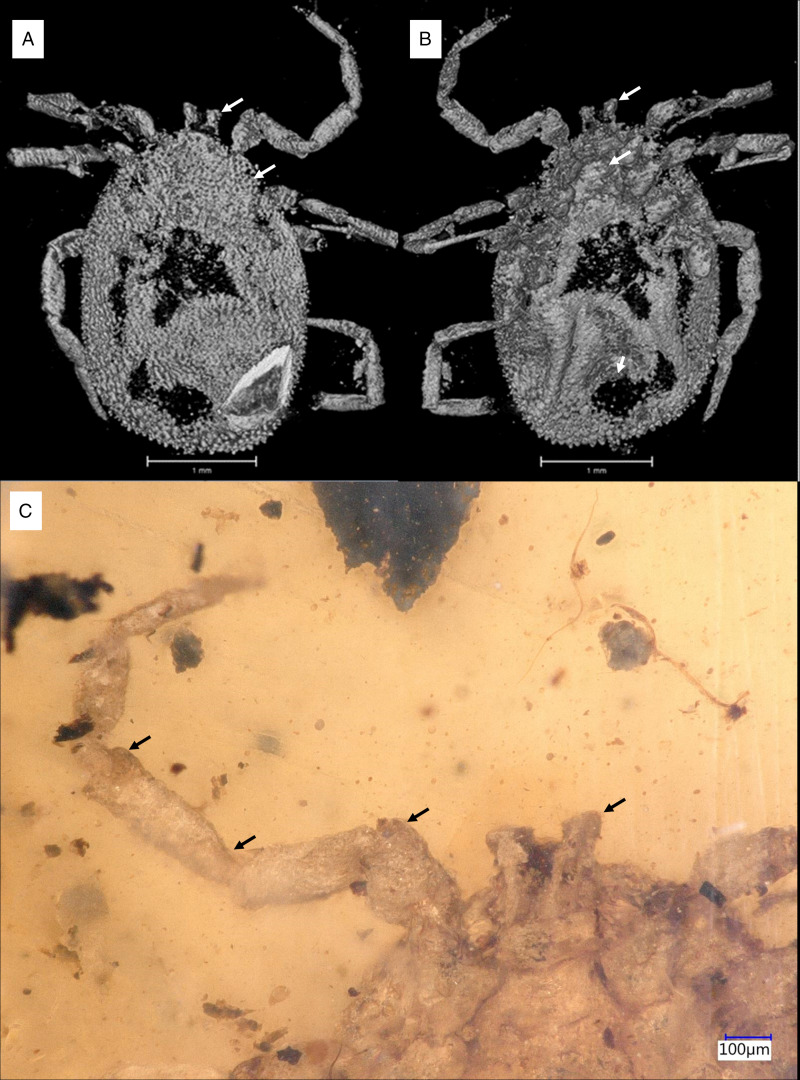
Holotype of *Deinocroton bicornis* sp. nov., collection no. B-4839, from Late Cretaceous (ca. 100 Ma) Burmese amber from Myanmar. (A) Dorsal view, arrow – palps, pseudoscutum. (B) Ventral view, arrow – palps, genital aperture, anal groove. (C) Ventral view, arrow – palps, detail of the leg joints.

*Idiosoma:* body subcircular; 2.95 mm length and 2.11 mm; dorsal and ventral surface with dense mammillae, without discs or sutural line between dorsal and ventral surface (Fig. 10A). Pseudoscutum in anterodorsal view is posteriorly broad V shape, 0.88 mm wide (measured in middle) and 1.02 mm long (from middle to edge); with large punctuations, cervical grooves absent (Fig. 10A). Genital aperture between coxae II (Fig. 10B). Anus close to the posterior body margin, anal groove slightly anterior the anus, encircle the anus, converging and closing, no reaching the posterior (Fig. 10B). Spiracular plate structure sub-circular in shape and consisting of a small depress concavity in the middle, entire plate arising from a depressed cuticular area. Eyes and festoons absent.

*Capitulum:* not visible in dorsal view, rectangular ventral 0.39 mm wide and 0.51 mm long, boarded by the coxae I; segment I 0.219 mm robust, segment II 0.186 mm, arched in appearance (creating a ventral concavity) and with a blade-like formation in the middle of the upper part, segment III 0.149 mm, arises from the segment II, segment IV 0.201 mm arises from the segment III (Fig. 10C and 3F).

**Figure 11.**
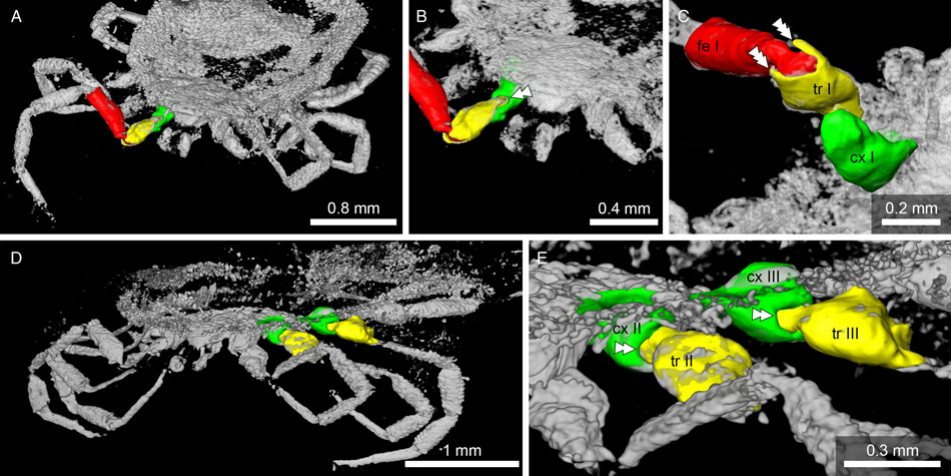
Micro-CT scan depicting the coxa/trochanter and trochanter/femur articulations in *Deinocroton* species. (A/C) *Deinocroton bicornis* sp. nov. (B-4839). (D/E) *Deinocroton copia* (BUB3319, specimen described in Chitimia-Dobler *et al*., 2022). Double arrowheads indicate notches, triple arrowheads indicate protrusions that restrict movement.

*Legs:* Coxae well developed; coxae I shows a single median spur and an external spur, coxae II, III and IV with an external spur; trochanter, femur, patella and tarsi articulations with notch-like processes. Dorsal and ventral edges of femur, genu and tibia riffled. Tarsi have very long claws and small round pulvillus (Fig. 10C). Articulations show some similarities to ball-and-socket joints. No real ‘ball’ present, but deep coxa/trochanter articulation with a distinct notch, and deep trochanter/femur articulation with 2 pronounced protrusions of cuticle framing a distinct notch (Fig. 11A–C).

*Chaetotaxy:* Long setae on the side and apical of the IV palp segments. Hair on the legs, especially on the leg joints, and long setae around the Haller's organ. Some spaced pits associated with setae.

#### *Deinocroton lacrimus* Chitimia-Dobler, Dunlop and Mans sp. nov.

http://zoobank.org/urn:lsid:zoobank.org:act:0342A9C3-B181-400E-83F4-FB0539E80C5A

*Etymology*: The species name *lacrimus* derive from the latin *lacrima* for teardrop referring to the distinct teardrop shape of B-4840.

*Material*: Holotype B-4840 (Berlin Museum). Burmese amber, Myanmar, Late Cretaceous (Cenomanian) (Supplementary video S8).

*Diagnosis*: *Diagnosis*: Legs long and strongly flattened laterally from trochanters to tarsi, without transverse ridges on leg segments. Palps very long, segments III and IV arise from the anterior segments. Segment II bent as an arch. Segment IV almost reach the basis capituli. Legs long and strongly flattened laterally from trochanters to tarsi, without transverse ridges on leg segments. Transverse genital aperture between coxae II.

*Description of the holotype male (B-4840):*

**Figure 12.**
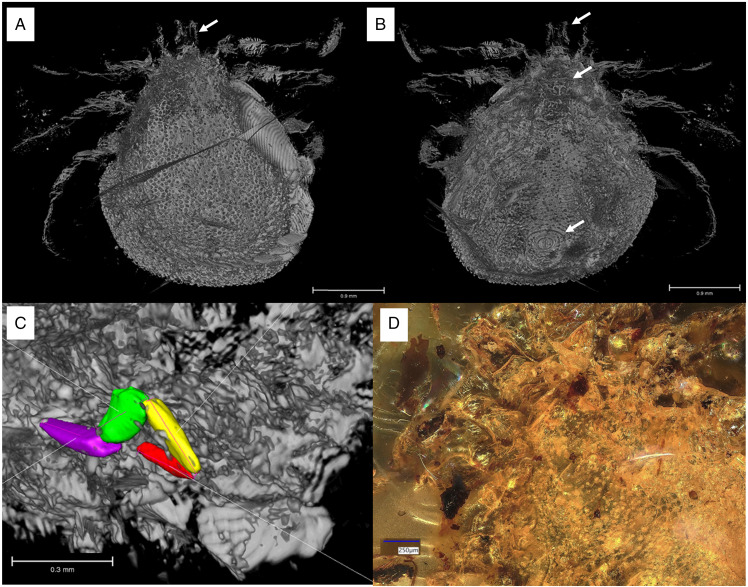
Holotype of *Deinocroton lacrimus* sp. nov., collection no. B-4840, from Late Cretaceous (ca. 100 Ma) Burmese amber from Myanmar. (A) Dorsal view, arrow – palps. (B) Ventral view, arrow – palps, anal groove. (C) Close up of palps. (D) Close up of coxae.

*Idiosoma:* body subcircular; 2.97 mm length and 2.59 mm; dorsal and ventral surface with dense mammillae, without discs or sutural line between dorsal and ventral surface (Fig. 11A and B). Pseudoscutum in anterodorsal view is posteriorly broad V shape; with large punctuations, cervical grooves absent (Fig. 12A). Genital aperture between coxae II. Anus close to the posterior body margin, anal valves well-defined, anal groove slightly anterior the anus, encircle the anus, converging and closing, no reaching the posterior body margin (Fig. 12B). Spiracle plate right structure sub-circular in shape and consisting of a small depress concavity in the middle, entire plate arising from a depressed cuticular area. Eyes and festoons absent.

*Capitulum:* not visible in dorsal view, segment I 0.202 mm, segment II 0.151 mm, arched in appearance (creating a ventral concavity) and with a blade-like formation in the middle of the upper part, segment III 0.186 mm, arises from the segment II, segment IV 0.192 mm longer and arises from the segment III.

*Legs:* Coxae not well visible; coxae I and IV no visible spur; trochanter, femur, patella and tarsi articulations with notch-like processes and without riffled dorsal and ventral line. Tarsus IV has very long claws and small round pulvillus.

*Chaetotaxy:* Few hairs on the legs, especially on the leg joints and some spaced pits associated with setae.

### Characters that differentiate Deinocroton species

*Deinocroton copia* derives mainly from the number and locality of the spurs found on the coxae. For coxae I, *D. bicornis* possess a single median spur and an external spur, and an external spur on the other coxae. *Deinocroton lacrimus* coxae without visible spurs. For coxae II, *D. copia* possess 2 spurs, medial posterior and distal anterior, while *D. draculi* possess 3 spurs, 2 medial and 1 distal anterior. Coxae III and IV, *D. draculi* presents 3 spurs, 2 basal, posterior and 1 medial anterior. *Deinocroton copia* present a single small anterior spur on coxae III and a small median blind spur on coxae IV. Genital aperture of *D. bicornis* and *D. lacrimus* between coxae II as in and *D. copia* and *D. draculi* males, as for the *D. draculi* female genital aperture is between coxae II and III.

### Ball-and-socket joints in the Nuttalliellidae compared to other families

The eponymous feature that defines the Nuttalliellidae is the presence of ball-and-socket joints, a feature seemingly unique to this family in the Parasitiformes (Mans *et al*., 2011). The question was raised whether this morphological trait may be present in other tick families, but obscured by external covering morphological structures such as a chitinous or membranous integument of the coxae and legs. To address this, micro-CT scans of the Nuttalliellidae (fossil and extant specimens), Ixodidae (extant) and Deinocrotonidae was performed. The Ixodidae are considered to be a proxy for the Argasidae, since previous studies indicated similar leg joints (Pavlovskiy, 1960; van der Hammen, 1983).

**Figure 13.**
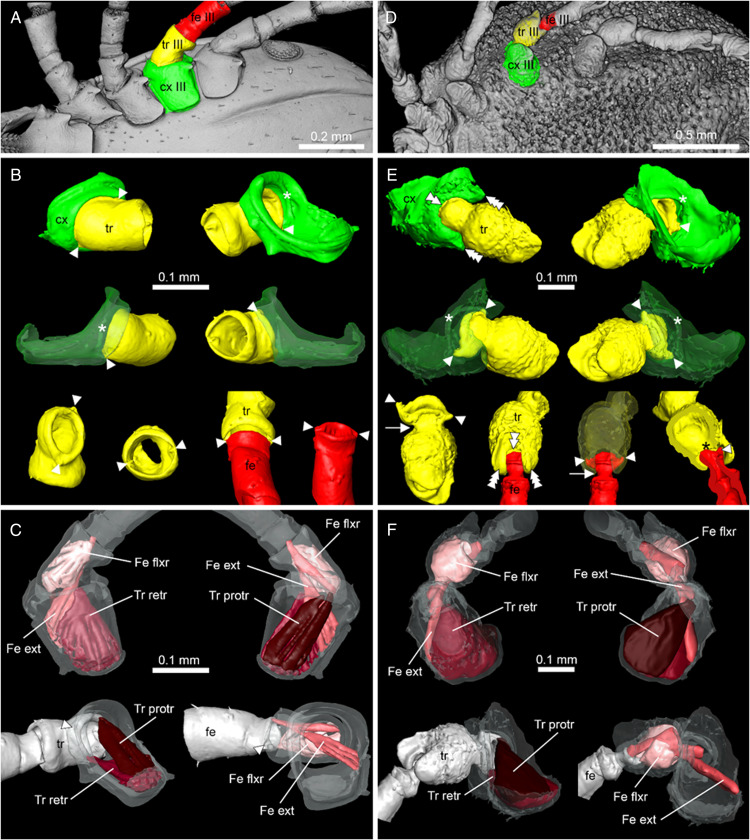
Comparison of the joint structures found in ixodid and *Nuttalliella* ticks. The left hand panels show a micro-CT scan of an extant *Ixodes ricinus* specimen and the right hand panels of extant *N. namaqua*. (A/D) Colour coding for the coxae, trochanter and femur. (B/E) Reconstruction of the articulation of coxae/trochanter and trochanter/femur. Single arrowheads indicate condyles, double arrowheads indicate notches, triple arrowheads indicate protrusions that restrict movement, the arrow indicates the neck region of trochanter and femur, and the asterisk indicates a rim of cuticle (*Ixodes*) which forms the socket in *Nuttalliella*. (C/F) Musculature in the coxa and trochanter. Images of *Nuttalliella* were mirrored for a better comparability.

In *I. ricinus* (Fig. 13A–C), the condyles (arrowheads) that form the actual bidesmatic bicondylar articulations between coxa/trochanter and trochanter/femur articulate superficially and thus can be seen from outside when looking at the 3D models of the joints. At the anterior margin of the coxa there is a small rim of cuticle (asterisk) that may function in supporting and guiding the movements of the trochanter. A protractor muscle moves the trochanter in an anterodorsal direction. A retractor moves the trochanter in a posteroventral direction. The femur is elevated dorsally by an extensor muscle that originates partially in the coxa and partially in the trochanter, while it is bent ventrally by a flexor muscle that has its sole origin in the trochanter.

In *N. namaqua* (Fig. 13D–F), the condyles (arrowheads) that form the bidesmatic ball-and-socket articulations between coxa/trochanter and trochanter/femur articulate deep in the proximal segment and thus cannot be seen from the outside. At the proximal margin of the coxa there is a pronounced rim of cuticle (asterisk) that forms a semi-circular socket that guides and supports movements of the trochanter. The cuticle of the coxa forms 2 protrusions (triple arrowheads) framing a deep notch (double arrowheads) that restricts the movement of the trochanter to a single plane. The joint between trochanter and femur is similar. The 2 condyles of the femur lie deep inside the trochanter segment. A rim of cuticle in the trochanter (asterisk) supports and guides movement of the femur. Like in the coxa, the trochanter shows 2 protrusions of cuticle (triple arrowheads) that frame a deep notch (double arrowhead), strictly restricting movement of the femur to a single plane. In both trochanter and femur, the width of the part that forms the 2 articulating condyles is similar to main part of the leg segments, while in between there is a narrower ‘neck’ region (arrow), which is the part that is moving within the notch of the proximal segment. This neck region is absent in the hard ticks. With regard to musculature, the situation is comparable to the hard ticks. A protractor and retractor move the trochanter in the anterodorsal and posteroventral direction, respectively, while the femur is lifted dorsally by an extensor and bent ventrally by a flexor muscle. Sites of origin and insertion of muscles are almost identical between *I. ricinus* ticks and *N. namaqua*.

In *D. bicornis* sp. nov. (Fig. 11A–C), articulations show some similarities to ball-and-socket joints found in *Nuttalliella* and *Legionaris*. Although no real ‘ball’ is present in either the trochanter or femur, articulations are located deeper than in hard ticks. The coxa shows a distinct notch (double arrowhead) that restricts movement of the trochanter to a single plane. The trochanter/femur articulation lies also deep, with 2 pronounced protrusions of cuticle (triple arrowheads) framing a notch that again restricts movement of the femur to a single plane. In *D. copia* (BUB3319, specimen described in Chitimia-Dobler *et al*., 2022) the coxa/trochanter articulation is similar (Fig. 11D and E). Again the coxa shows a notch (double arrowheads) that restricts movement of the trochanter.

## Discussion

### Generalists and extinction

It was previously suggested that *N. namaqua* is a host generalist species and that this allowed survival through multiple extinction events (Mans *et al*., 2014). It would seem from the fossil record that a large number of species or lineages from the Nuttalliellidae did not survive mass extinction events. It is therefore likely that some of these were more host specific and perhaps associated with hosts that also became extinct. In the context of the fossils, it is likely that these species parasitized dinosaurs and that the K–Pg mass extinction event resulted in the extinction of the majority of species in the Nuttalliellidae. As such, the Nuttalliellidae may only now be depauperate, but were historically a species and genus rich lineage (see Barker *et al*., 2014 for speculation on this). Nuttalliella lineages that survived may have been those that showed host generalist behaviour (Mans *et al*., 2014).

### Historical distributions and ecological niches

The presence of *Nuttalliella* in Burmese amber has several interesting implications. The first is that its historical distribution was much larger than its current distribution that is limited to East and southern Africa. In this regard, the Gondwanean affinities of Burmese amber fossils suggest that fossils probably derived from northern Australia and rifted on the Burmese terrane ~150 MYA en route to Asia. Formation of fossils occurred ~100 MYA somewhere in the Indian Ocean (Westerweel *et al*., 2019). As such, *Nuttalliella* would have been present in northern Australia at the time of rifting. The divergence of *Nuttalliella* from the other tick families have been estimated at ~270 MYA based on molecular clock analysis (Mans *et al*., 2019). Its origins were proposed to have been in the Karoo basin of southern Africa (Mans *et al*., 2011). The current finding of *Nuttalliella* in Burmese amber would suggest that this genus was distributed from southern Africa to Northern Australia at the time of rifting. Africa, Antarctica and Australia were a single landmass 150 MYA, with what is today southern Australia linked to Antarctica and northern Australia at the eastern-most edge of Australia (McLoughlin, 2001). Migration from southern Africa to northern Australia would have occurred *via* Antarctica to southern Australia to northern Australia. It raises the interesting prospect that extant *Nuttalliella* may still be found in Australia (see Barker *et al*., 2014 for speculation on this).

The extant ecological niches of *N. namaqua* is considered xeric (Mans *et al*., 2011), while the sister group to the Ixodida, the Holothyrida has an extant geographic distribution in the tropical archipelagos from Melanesia to the Seychelles (Holothyridae), Australia (Allothyridae) and Caribbean (Neothyridae). It was suggested that Holothyrida had a Gondwanean origin with a geographic distribution that ranged across Gondwanaland, with localized extinction events leading to the current day distributions (Lehtinen, 1991, 1995). Conversely, it was suggested that *N. namaqua* originated when the Karoo basin appeared in southern Africa allowing for a niche adaption to a drier environment (Mans *et al*., 2011). The presence of *Nuttalliella* in the tropical amber forest raise the interesting prospect that *Nuttalliella* originated in the same tropical niche as the sistergroup to ticks, the Holothyrida. In this case, the extant Afrotropical distribution that seems limited to the xeric regions would have been a specific adaptation. Alternatively, it suggests that extant *Nuttalliella* may have a much wider distribution than currently recognized and may also be found in tropical regions of former Gondwanaland.

### Ball-and-socket joints in the Nuttalliellidae compared to other families

The Nuttalliellidae clearly have ball-and-socket-like joints as depicted in both extant and fossil specimens. The extant specimen also clearly shows that, for the coxa-trochanter and the trochanter-femur articulation, the ball is seated deep within the socket and has 2 condyles that would allow a hinge-like movement with a single-degree of freedom for all 4 walking legs. The socket is formed by a distinct rim that supports the ball. However, in the first pair of legs, coxa I is separate from other coxae and much longer than coxae II-IV. The coxa-trochanter and trochanter-femur joints are both bidesmatic.

The Ixodidae present the classical bicondylar bidesmatic coxa/trochanter, and trochanter/femur joints, as reported before (van der Hammen, 1983). This type of joint structure does not resemble at all a ball-and-socket joint and is different from what is observed in the *Nuttalliella*, because the actual site of articulation (condyles) is situated superficially and not deep within the proximal segment. Deinocrotonidae, on the other hand, show articulations that are more similar to *Nuttalliella*. Although no real ‘ball’ is present in either trochanter and femur, articulations lie deep in the proximal segment, and deep notches are present in both coxa and trochanter that restrict movements of the distal segment. *Legionaris* nov. gen. shows a similar ball-and-socket joint such as *Nuttalliella*. It should be considered that all members of the Nuttalliellidae do not have a true ball-and-socket joint in the functional sense (like human shoulder or hip joints, which allow multiple degrees of freedom of movement), since free movement is not possible given the condyles. As such, *Deinocroton*, *Legionaris* and *Nuttalliella* may be considered to present a general type of joint that differ from argasids and ixodids that is characterized by distinct condyles that insert deep into the basal articulations.

### Palps in the nuttalliellidae

*Nuttalliella* genus palps are characterized by a small segment I, massive segments II and medium III segments that arise from segment II, and segment IV arise from segment III. *Legionaris* has a massive segment I and segment II and III are robust, arise from the anterior segments divided in 2 anteriorly, segment III bend, and segment IV arises from the segment III and looks like in *Nuttalliella*. *Deinocroton* segment I longer than wide, segment II arched in appearance creating a central concavity and with a blade-like formation in the middle of the upper part, segment III arises from the segment II, segment IV arises from the segment III. Arising of segments II-IV from the anterior segment is considered a specific characteristic for all 3 genera and together with ‘ball-and-socket-like’ joints configuration suggest that these genera are evolutionary related and share defining characters that may be considered an independent evolutionary lineage.

### Relationships between the Deinocrotonidae and Nuttalliellidae: One family?

The presence of ‘ball-and-socket-like’ joints in *Deinocroton* and *Legionaris* gen. nov. not only supports the sister relationship of the Deinocrotonidae to the Nuttalliellidae (Peñalver *et al*., 2017) but also suggest a much closer relationship between the 2 families. With the addition of the genus *Legionaris* gen. nov. to the Nuttalliellidae, we consider that the differences between these families do not warrant separate family status anymore, but would warrant inclusion of the Deinocrotonidae in the Nuttallielliedae. This would reduce the current recognized tick families to 3 extant families (Argasidae, Ixodidae and Nuttalliellidae) and one extinct family (Khimairidae). The defining characters for the family would then be the presence of a pseudo-scutum/pseudo-conscutum, convoluted structure of the integument, sub-terminal mouthparts with arising of segments II–IV from the anterior segment and leg joints that extend deep into the preceding cavity, either as ball-and-socket (*Nuttalliella*, *Legionaris*) or approximating a socket joint (*Deinocroton*). It may be considered that the 3 genera presents similar or less morphological differences than observed for members of either the Argasidae or Ixodidae (Klompen and Oliver, 1993; Klompen *et al*., 1997). Given that *Deinocroton*, *Legionaris* and *Nuttalliella* are considered to be an evolutionary lineage to the exclusion of the Ixodidae or Argasidae it makes taxonomic and evolutionary sense to unite them into one family, the Nuttallielliedae.

### One family, different genera

The differentiation of the argasid and ixodid families are relatively straightforward. The presence or absence of a scutum, the ‘hard’ *vs* ‘soft’ integument and the terminal *vs* ventral position of the basis capitulum in ixodids and argasids, respectively. Using these same criteria, it is clear that *Deinocroton*, *Legionaris* and *Nuttalliella* would fall within their own unique but the same family, the Nuttalliellidae. As such, these genera share the presence of a pseudoscutum, a ‘soft’ integument and the sub-terminal position of the basis capitulum. This already differentiate them from the other families. In addition, these genera share leg joints with deep articulation within the joint, giving them a ‘ball-and-socket’ appearance, even though it is clear that this is an artefact of the deep articulation with none of the genera having ‘ball-and-socket’ joints with free rotation. Another unique morphological characteristic is on palps, segment II and IV arising from the anterior segments. Within this family the genera can be readily differentiated by the following characters: *Nuttalliella* genus has a classical ball-and-socket articulation with a round distal region going into the socket; *Legionaris* has a condyles joints with an ovoid distal region, while *Deinocroton* has a round-ovoid distal region. The 3 genera differed also on palps: *Nuttalliella* palps have a triangular aspect in dorsal view; segment II massive; segments III and IV arise from the anterior segments. *Legionaris* segment I massive; segments II, III and IV arise from the anterior segments; segment III bent direct on the arising point from the segment II. *Deinocroton* palps are very long, segments III and IV arise from the anterior segments; segment II bent as an arch; segment IV almost reach the basis capituli. Another important difference is in the legs: *Nuttalliella* has long, slender, beaded legs; *Legionaris* has very robust legs; *Deinocroton* has long and strongly flattened laterally legs from trochanters to tarsi and sometimes with transverse ridges on some all or some leg segments. Genital aperture can also be considered in differentiate the 3 genera: *Nuttalliella* has a transverse slit (external margins of lips dentate in male) between coxae I on male and coxae II in female; *Legionaris* has aperture bipartite in appearance, with bulging triangular forms and a narrow central flat line between, flanked around by an oval groove, to the posterior level of coxa I; *Deinocroton* has a transverse genital aperture between coxae II on male and between coxae II and III on female (according to Peñalver *et al*., 2017).

## Conclusion

The only tick representatives not found in Burmese amber to date are the Argasidae. Conversely, a remarkable assemblage of tick fossils of both extant and extinct families and genera have been revealed in Burmese amber including the extinct genera *Compluriscutula*, *Cornupalpatum*, *Deinocroton*, *Khimaira* and *Legionaris*, respectively. Fossils for various extant genera has also been found, including the prostriate *Ixodes* and the metastriate *Amblyomma*, *Archaecroton*, *Bothriocroton*, *Haemaphysalis* and now the *Nuttalliella*. These lineages all have minimum dates of origin of at least 100 Ma and if the Gondwanean hypothesis is correct would extent the minimum date of origin to ~150 Ma. It also suggests that the origin of ticks may lie somewhere on the Gondwanean continent. In addition, description of new species and genera for *Deinocroton*, *Legionaris* and *Nuttalliella* and their leg joint articulation allow the unification of 3 genera into one family, the Nuttalliellidae.

## Supporting information

Chitimia-Dobler et al. supplementary material 1 Chitimia-Dobler et al. supplementary material

Chitimia-Dobler et al. supplementary material 2 Chitimia-Dobler et al. supplementary material

## Data Availability

All data are available in publication and supplementary material. Raw data may be obtained from the University of Vienna.

## Supplementary material

The supplementary material for this article can be found at https://doi.org/10.1017/S0031182024000477.
