# Supplementary material for: Nuttalliellidae in Burmese amber: implications for tick evolution
Source: Parasitology. 2024 Apr 16;151(9):891–907. doi: 10.1017/S0031182024000477 (PMC11770530; doi:10.1017/S0031182024000477)
Supplement: Chitimia-Dobler et al. supplementary material 1 — Chitimia-Dobler et al. supplementary material [file S0031182024000477sup001.pdf]

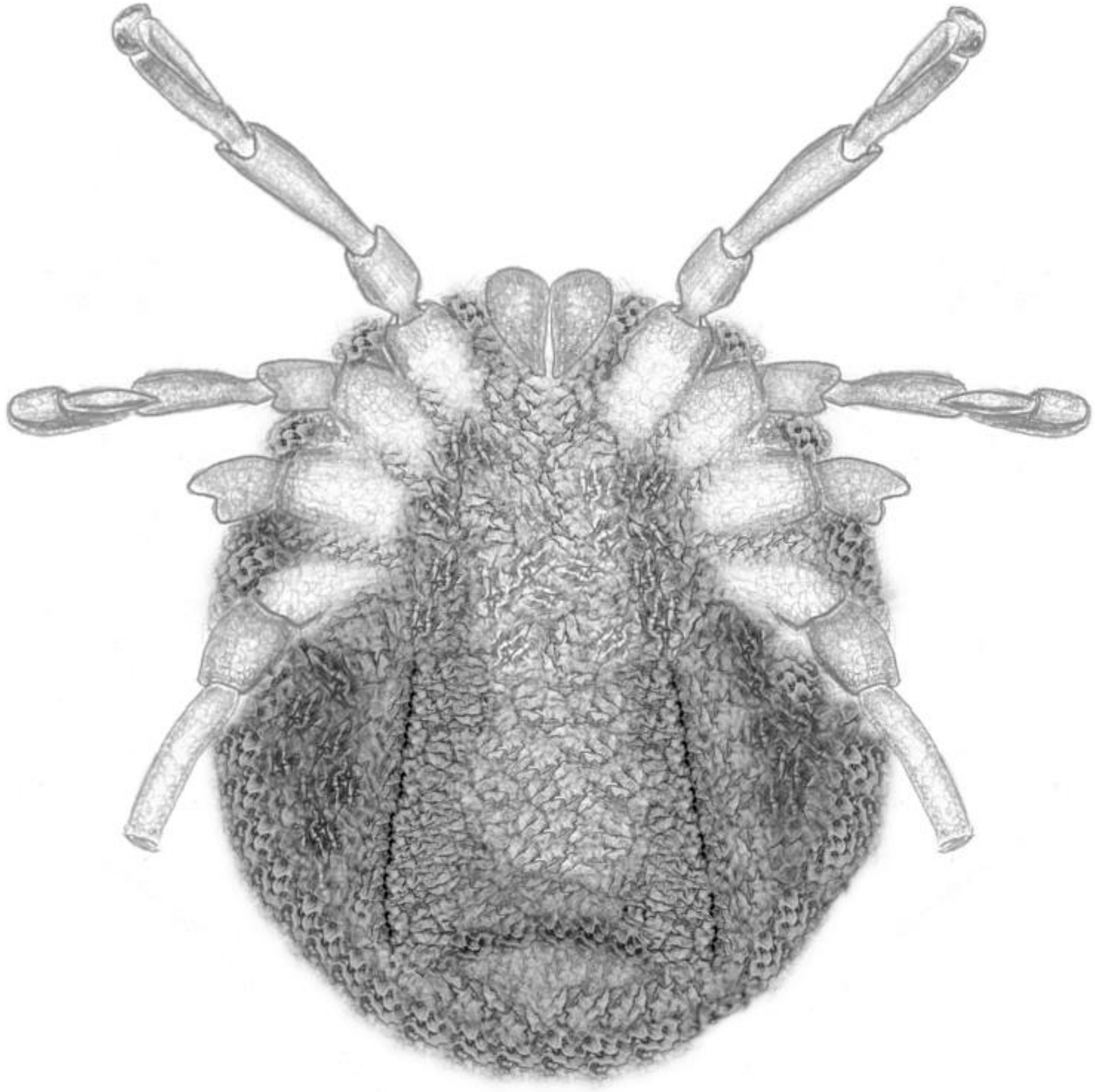

Figure S1: Artist's rendering of ventral side of *Nuttalliella odyssea* sp. nov., fossil collection no. B-4863. Ronel Pienaar.

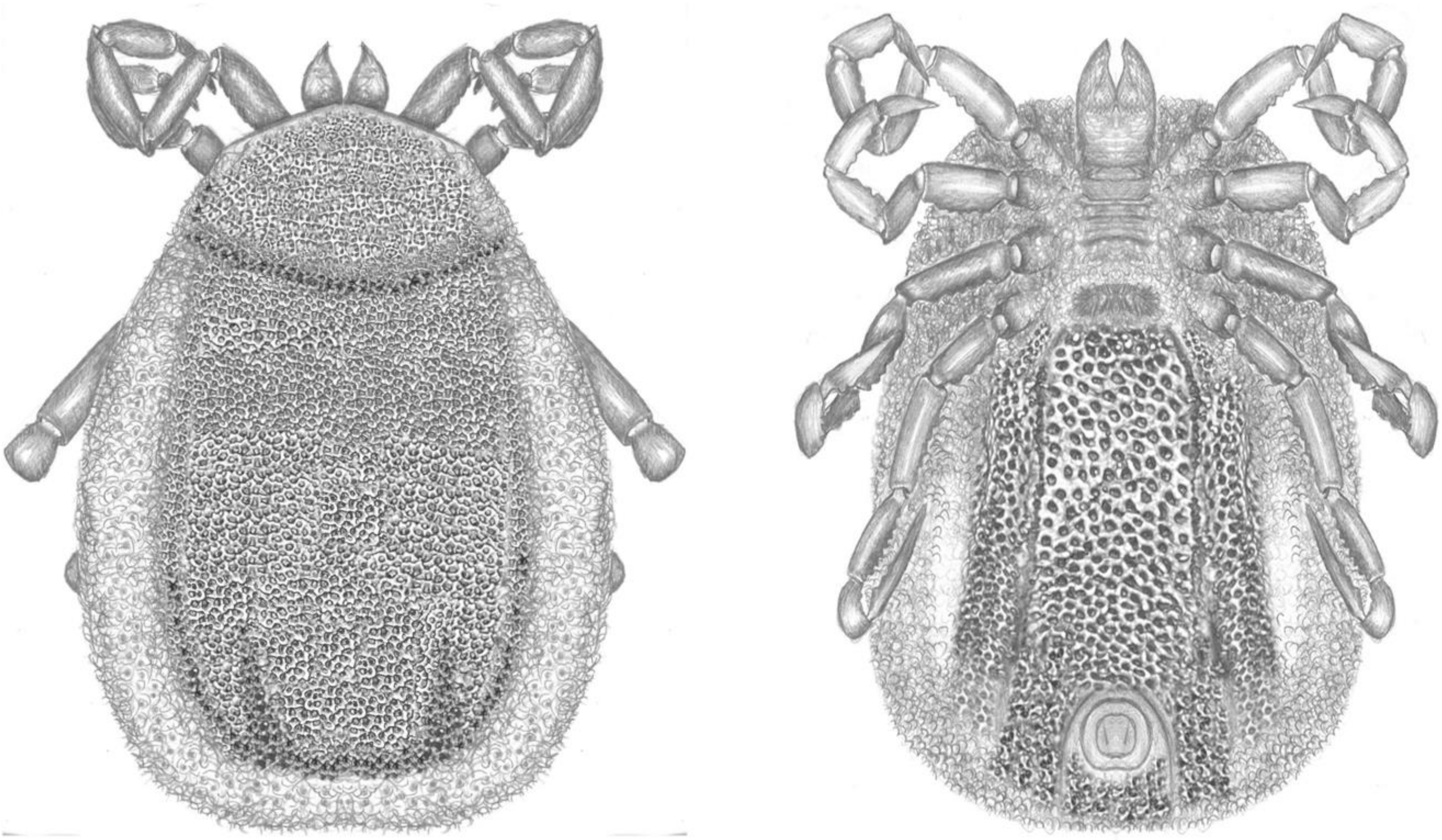

Figure 2S: Artist rendering of the male *Nuttalliella placaventrala* sp. nov., B-4862. Ronel Pienaar.

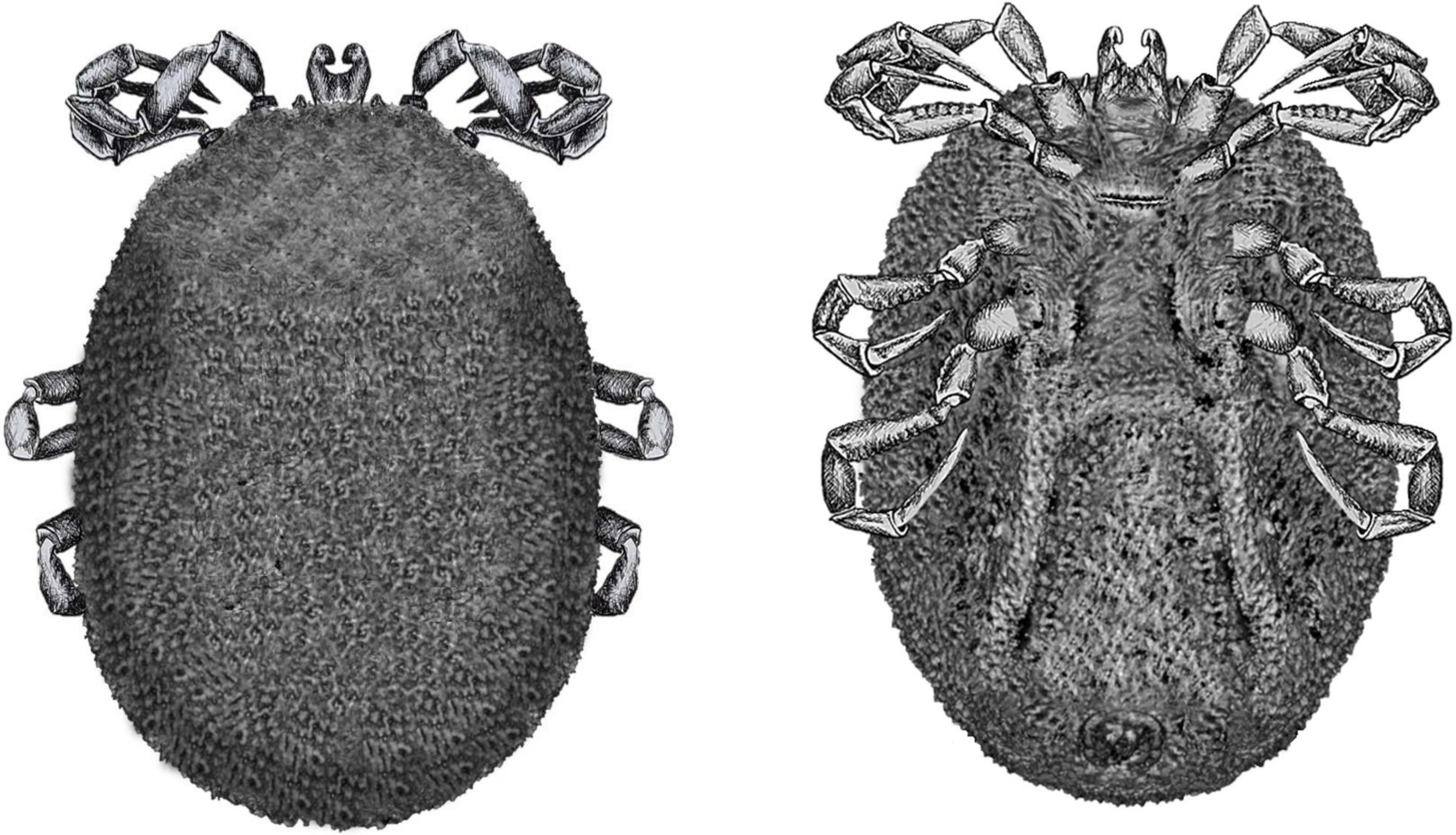

Figure S3: Artist's rendering of ventral side of *Nuttalliella tropicasylvae* sp. nov., fossil collection no. B-4863. Ronel Pienaar.

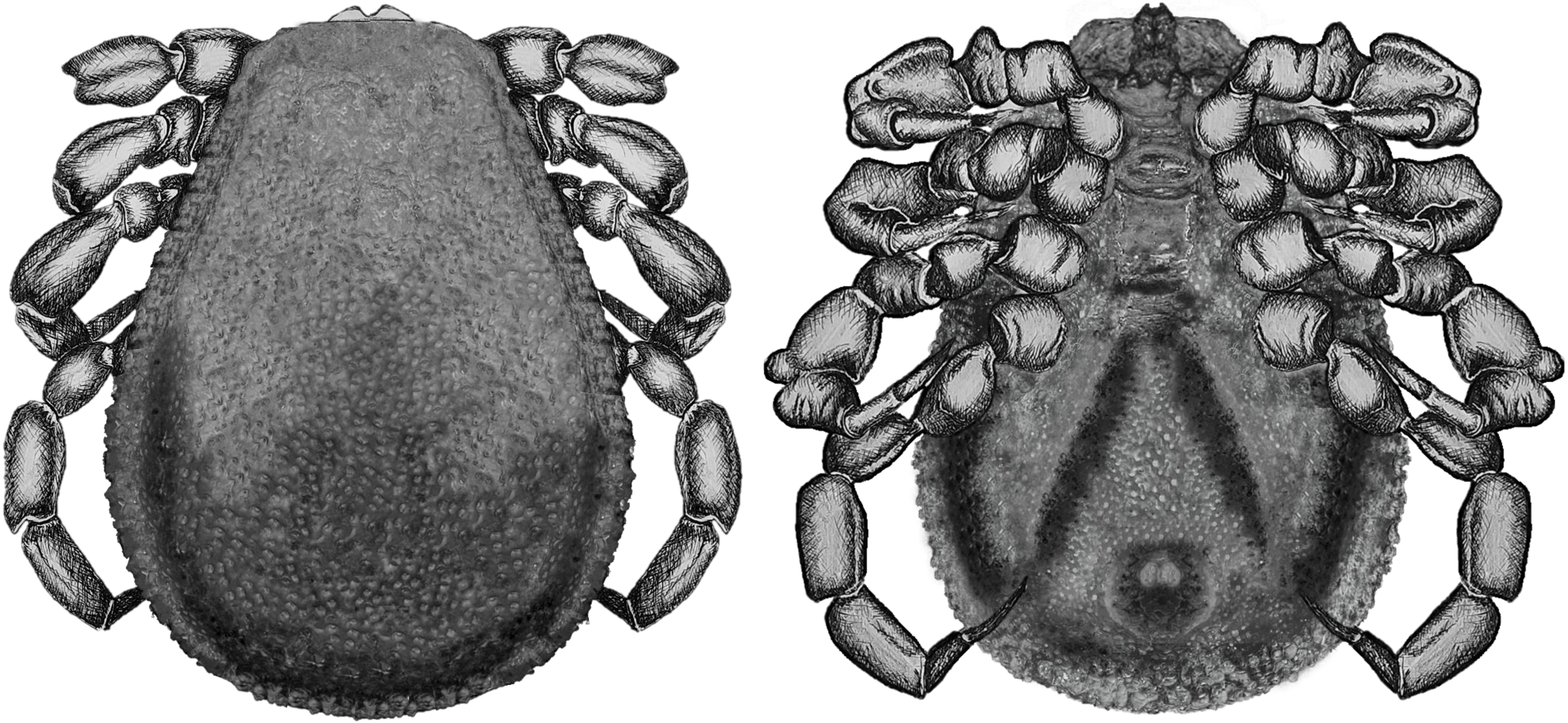

Figure S4: Artist's rendering of ventral side of *Legionaris robustus* sp. nov., collection no. B-4891. Ronel Pienaar.

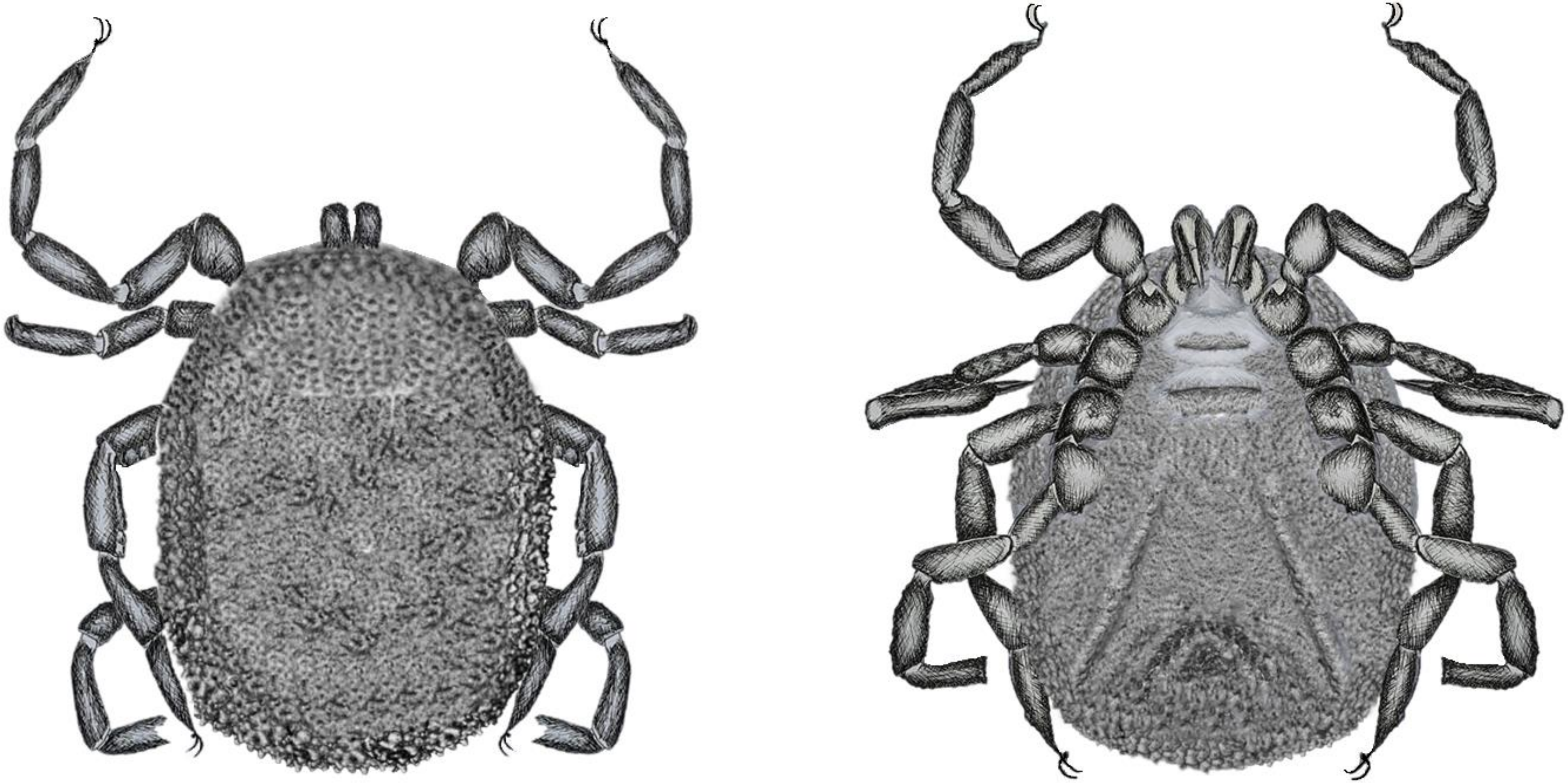

Figure S5: Artist's rendering of ventral side of *Deinocroton bicornis* sp. nov., collection no. B-4839. Ronel Pienaar©.

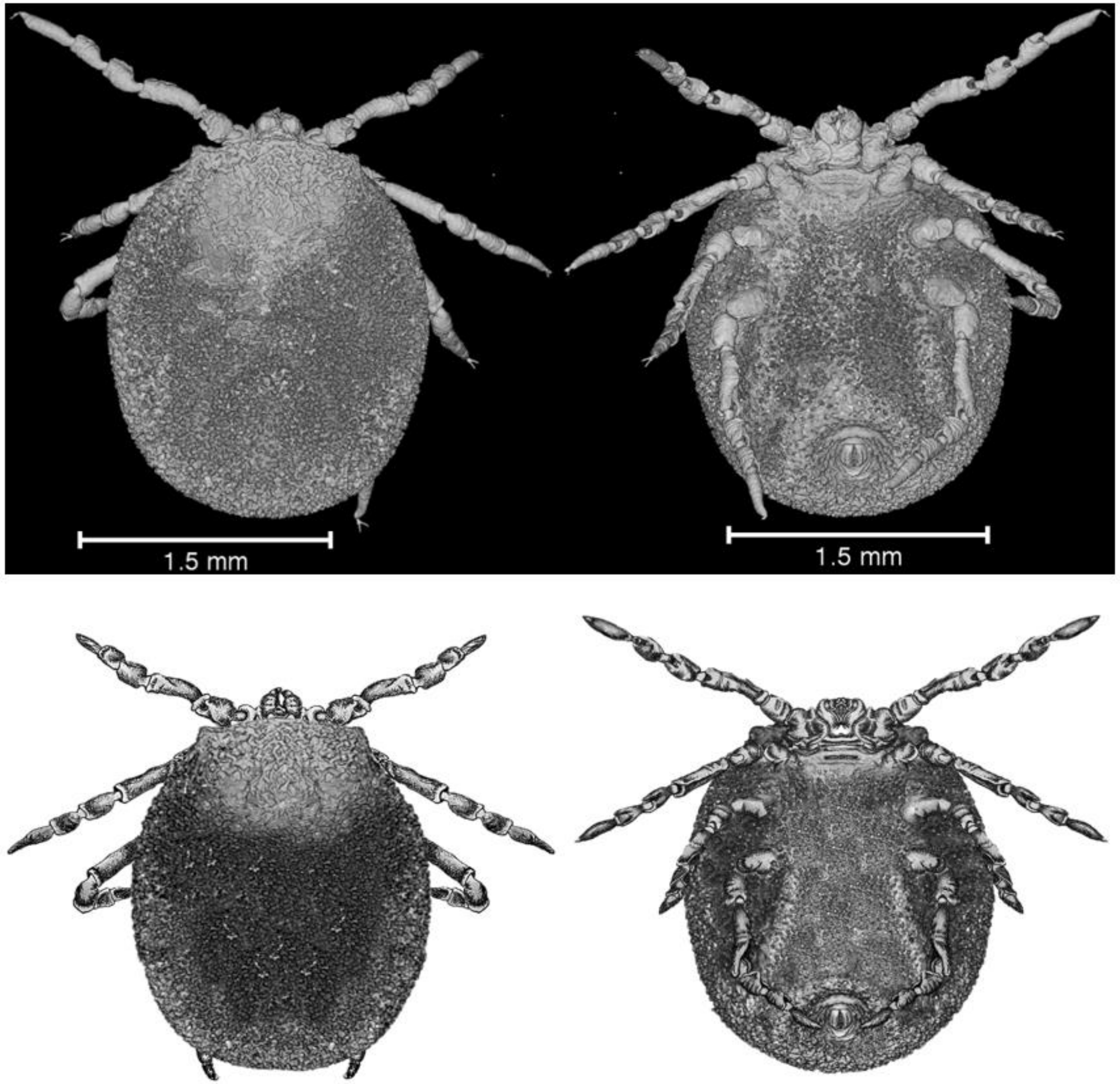

Figure S6: Extant *Nuttalliella namaqua*. Indicated in the top panel are micro-CT scans of the dorsal and ventral views of a female *N. namaqua* as well as an artist depiction of the same features to allow comparison with similar images of fossil specimens.
